# Infant feeding practices in Bhaktapur, Nepal: a cross-sectional, health facility based survey

**DOI:** 10.1186/1746-4358-7-1

**Published:** 2012-01-10

**Authors:** Manjeswori Ulak, Ram K Chandyo, Lotta Mellander, Prakash S Shrestha, Tor A Strand

**Affiliations:** 1 CHRP, Department of Paediatrics, IOM, Nepal; 2 Centre for International Health, University of Bergen, Norway; 3 Department of Community Medicine, Kathmandu Medical College, Nepal; 4 Department of Paediatrics, Göteborg University, Sweden; 5 Division of Infectious Disease Control, Norwegian Institute of Public Health, Oslo, Norway; 6 Medical Microbiology, Innlandet Hospital Trust, Lillehammer N-2629, Norway

**Keywords:** Exclusive breastfeeding, mixed feeding, infant, Nepal

## Abstract

**Background:**

Promotion of proper breastfeeding practices for the first six months of life is the most cost-effective intervention for reducing childhood morbidity and mortality. However, the adherence to breastfeeding recommendations in many developing countries is not satisfactory. The aims of the study were to determine breastfeeding and infant feeding patterns at nine months of age and to assess factors influencing exclusive breastfeeding practices.

**Methods:**

In Bhaktapur, Nepal, we carried out a cross-sectional survey of 325 infants who came for measles vaccination at the age of nine months. Mothers were interviewed on details regarding feeding of their child and health since birth.

**Results:**

Three quarters of all mothers reported that they did not receive any information on breastfeeding during the antenatal visit. Two hundred and ninety five (91%) mothers gave colostrum and 185 (57%) initiated breastfeeding within one hour of delivery. The prevalence of exclusively breastfeeding at 1, 3 and 6 months were 240 (74%), 78 (24%) and 29 (9%), and partial feeding was initiated in 49 (15%), 124 (38%) and 257 (79%) babies, respectively. The main reason, according to the mother, for introducing other foods before six months of age was insufficient breast milk. In logistic regression analyses, mother's knowledge on how long child should be given only breast milk and not living in joint families were associated positively with exclusive or predominant breastfeeding for four months or beyond.

**Conclusions:**

Despite the high proportion of mothers who initiated breastfeeding immediately after birth, continuation of exclusive breastfeeding for up to six months was not common. Very few mothers received any information on breastfeeding during the antenatal visit, indicating a need for counseling on exclusive breastfeeding. Possible options for this counseling could be during antenatal visits and at regular clinic visits for vaccination.

## Background

Adequate nutrition during infancy is crucial for child survival, optimal growth and development throughout life [1]. The World Health Organization (WHO) recommends exclusive breastfeeding (EBF) for the first six months of life [2]. After six months, infants should receive nutritionally adequate and safe complementary foods while continuing to be breastfed until the age of two years or beyond. The benefit of EBF for growth, immunity and prevention of illness in young infants is undisputable [3,4]. It has been postulated that 13% of the current under five mortality rate could be averted by promoting proper breastfeeding practices [5], which is seemingly the single most cost effective intervention to reduce child mortality in resource-constrained settings such as in Nepal [6]. The importance of EBF for optimal growth and development, irrespective of country of residence, is also reflected in the recent WHO growth standard for children [7]. Introduction of foods other than breast milk before six months of life is not only undesirable, but could also be harmful [8]. These foods not only displace nutritious mother's milk, but also serve as a vehicle for infectious pathogens that can lead to severe illness. Despite well-established guidelines for promotion of EBF, the adherence to EBF is quite low in many settings [9-11]. Childhood malnutrition and growth faltering affects more than half of children under five in developing countries, and usually starts during infancy, possibly due to improper breastfeeding and mixed feeding practices [12].

The mean total duration of breastfeeding in Nepal, like most other low and middle income countries, is long and usually more than two years [13], but data on EBF up to six months of age as well as descriptions of mixed feeding practices are scarce. Information on breastfeeding practices and the factors influencing them is important for successful campaigns. Hence, we undertook a measles vaccination clinic-based cross-sectional survey in Bhaktapur, Nepal. We used a structured questionnaire for an in-depth interview on breastfeeding and complementary feeding practices since birth.

## Methods

### Study area and population

This study was conducted in a vaccination clinic at Siddhi Memorial Children's Hospital in Bhaktapur, Nepal. Bhaktapur is located 15 km east of the capital Kathmandu. It has a total population of approximately 75,000 [14], predominantly of the Newar ethnic group and mostly farmers, semi-skilled or unskilled labourers and daily wage earners. The vaccination clinic in the hospital provides service twice a week with an average flow of 110 children per week. This is one of the most widely used vaccination clinics inside the municipality area in Bhaktapur [15]. Our experience is that vaccination clinics in our settings mainly focus on immunization and do not undertake any breastfeeding counselling.

### Study design and participants

This cross-sectional survey was carried out from August to December 2007 on a convenient sample of infants coming for measles vaccination, which is given as soon as possible after nine months of age in Nepal. This is the last regular contact with health centres for free vaccination by the government, and at a time when it is feasible to collect data on breastfeeding and complementary feeding practices. Sample sizes were calculated using standard formulas available at Open Source Epidemiologic Statistics for Public Health [16]. The sample size was calculated assuming that the proportion who were exclusively breastfeed at six months of age was 50%. An absolute precision of 6%, (corresponding to a confidence interval of 50% ± 6%) required a sample of nearly 300 mother and infant pairs. For outcomes with prevalence different from 50%, the absolute precision would be higher for this sample size.

Infants were included in the study if they lived in the study area and completed nine months of age. Infants with specific feeding problems (cleft lip or palate, congenital heart disease, severe illness during neonatal period or delayed developmental milestones) were excluded from the study. All children coming for measles vaccination during the study period were screened and enrolled if eligible. The ethical board of the Institute of Medicine, Tribhuvan University in Katmandu, Nepal approved the study. We took verbal consent from parents or caretakers before administrating the questionnaire.

Field workers, who were trained by the first author (MU) on anthropometry measurement and breastfeeding, administered a structured questionnaire on breastfeeding practices and on child morbidity (Additional file 1). These field workers speak the local language, have experience from similar studies, and are accordingly familiar with these types of questionnaires. The first author also supervised about 10% of the interviews conducted by the field workers and carried out pilot testing of forms in 50 infants prior to the study. Children were weighed on a UNICEF electronic pediatric scale (SECA, Germany) with 100 g sensitivity. Length was measured using a locally made wooden board with an accuracy of ± 0.1 cm. Weight-for-length, length-for-age and weight-for-age Z-scores were calculated using the recent WHO growth charts [7].

### Definition of breastfeeding categories

We defined breastfeeding according to the recent WHO guidelines [17], which categorize into three groups; exclusive, predominant and partial breastfeeding, and focused on the entire period since birth as described by Labbok et al [18].

#### Exclusive breastfeeding

The infant had received only breast milk from his/her mother or a wet nurse, or expressed breast milk and no other liquids or solids with the exception of drops of syrup consisting of vitamins, mineral supplements or medicines.

#### Predominant breastfeeding

The infant's predominant source of nourishment had been breast milk. However, the infant may also have received water and water-based drinks like tea and local herbal drops.

#### Partial breastfeeding

When infant's feeding included non-breast milk foods such as animal/powdered/condensed milk and/or solid/semi-solid food (i.e. cereals, vegetables, fruits, lentils or meat).

### Data entry and statistical analysis

All forms were checked manually for completeness and consistency. The forms were double entered into a Visual Fox Pro database with consistency, logic and range checks. Descriptive statistics include mean and standard deviation of continuous variables and proportions for categorical variables. Chi-square test was used if the variables were categorical and t-tests were used for continuous variables. Exclusive or predominant breastfeeding for 4 months or beyond was used as a dependent variable in logistic regression analysis. Crude and adjusted odds ratios and the corresponding 95% confidence intervals were calculated. A p value of less than 0.05 was considered statistically significant. The statistical analyses were undertaken using Stata^®^, version 9.2.

## Results

### Baseline features

A total of 2,166 children attended in the vaccination clinic during the study period for different vaccines and 392 for measles vaccination. Sixty seven children could not be enrolled because they were from outside the study area or met other exclusion criteria. We enrolled 325 infants, of which 205 (63%) were living in joint families, 177 (54.5%) were male and 185 (57%) first born. One third of the families of the infants were staying in rented rooms. A total of 273 (84%) infants were 9 months and the remaining were 10 months of age. The age range of the mothers was 17-45 years and 267 (82%) were 21-30 years of age. The other general features of children and their parents are presented in Table 1.

**Table 1:** General characteristics of household, parents and infants in Bhaktapur, Nepal

Characteristics	N (325)	%
**Gender of child**		

Male	177	54.5

Female	148	45.5

**Age of mother**		

25 years and younger	175	53.8

> 25 years	150	46.2

**Birth weight < 2.5 kg (n = 294)**		

Yes	38	13

No	256	87

**Type of delivery**		

Normal	268	82.5

Cesarean section	57	17.5

**Education of mother**		

Illiterate or primary	148	45.5

Up to grade 10 or above	177	54.5

**Occupation of mother**		

No work or agriculture	219	67.4

Working	106	32.6

### Knowledge and practices influencing the breastfeeding pattern

All but seven women said that they had visited antenatal clinics during the last pregnancy. However, only one quarter of them reported that they received some information on breastfeeding during their antenatal visit (Table 2). A total of 257 infants (79%) were introduced to other foods (semi/solid or animal milk) before six months of age and the main reason was assumed insufficient breast milk production. Working outside home was reported by one third of mothers, and half of them also mentioned that this was the reason for not exclusively breastfeeding. The decision to give other foods before six months of age was taken by the mother herself for 136 infants (42%) and grandmothers decided to do so for 94 infants (29%).

**Table 2:** Knowledge and factors influencing breastfeeding practice in Bhaktapur, Nepal

Characteristics	N	%
**Information on breastfeeding during antenatal visit**		
Yes	83	25.5
No	235	72.3
Did not have antenatal visit	7	2.2
**How long mother thinks only breast milk will be enough for child**		
< 6 months	93	28.6
6 months or more	160	49.2
Do not know	72	22.2
**Reason for introducing other food before 6 months ^1^**		
Crying/hungry	75	29.2
No enough breast milk	132	51.4
Mother's illness	8	3.1
Working mother and others	42	16.3

^1 ^Based on 257 infants who had other foods introduced before 6 months of age

### Breastfeeding and mixed feeding practice

**Figure 1:**
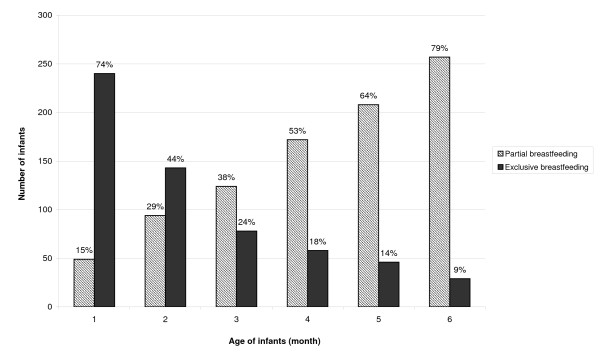
**Proportion of exclusive and partial breastfeeding patterns among infants in Bhaktapur, Nepal**.

Almost all infants (99.7%, n = 324) were continuing to breastfed at the time of interview. The patterns of prelacteal and colostrum feeding practices and initiation of breastfeeding practices after birth are presented in Table 3. The prevalence of EBF at 1, 3 and 6 months was 240 (74%), 78 (24%) and 29 (9%), whereas partial feeding was introduced during these periods by 49 (15%), 124 (38%) and 257 (79%) of mothers, respectively (Figure 1). Water and local herbal drops (*janamghuti*) were the two most commonly introduced drinks in first two months, followed by local semisolid porridge (*lito*), which was given to half of the infants during first four months (Table 4), and powder milk was given to 101 (31%) children.

**Table 3:** Breastfeeding initiation and prelacteal feeding patterns in Bhaktapur, Nepal

Breastfeeding practice (n = 325)	N	%
Any breastfeeding	324	99.7
Prelacteal feed given	55	17
Breastfeeding started within 1 hour	185	57
Breastfeeding started within 24 hours	286	88
Colostrum given	296	91

**Table 4:** Mixed feeding practices among 325 infants in Bhaktapur, Nepal^1^

Food/drinks	% of children reported to receive regularly^2^	Median month of starting	Interquartile range
Water	94	2	1, 4
Herbal drops (*janamghuti*)^3^	71	1	1, 1
Nuts, dates^4^	42	2.5	1, 5
Local porridge/cerelac	72	4	2, 6
Powder milk	31	3	1, 5
Animal milk	49	6	4, 8
Biscuits	73	6	5, 7
Rice/lentils	93	6	6, 7
Potato/vegetables	79	6	6, 7
Breads (home-made or readymade)	40	7	6, 8
Fruits	35	7	6, 8
Noodles	13	8	6, 8
Eggs	47	7	6, 8
Meats	28	7	6 ,8

^1 ^Food given separately, mixed with others or in the form of soup or juices

^2 ^Three or more times a week

^3 ^Commercially available liquid preparations

^4 ^Usually given in mashed form by licking finger

### Anthropometry and breastfeeding practice

The mean weight and length of infants were 8.3 kg (SD 1.1) and 69.8 cm (SD 3.1) respectively. The mean weight and height of infants who were EBF for six months were not significantly different from those who were not. However, those infants who were introduced to semi-solid foods during the first two months of life were significantly shorter than those who were not (70.2 cm vs. 69.1 cm, p = 0.006).

### Parent's education, living and perinatal conditions and breastfeeding practices

Infants living in joint families were more likely to have received herbal drops than infants from nuclear families (84% vs. 64%, p < 0.001) or families staying in their own home than in those staying in rental homes (84% vs. 57%, p < 0.001). Similarly, these infants were more likely to have been introduced to semi-solid foods in the first two months (34% vs. 20%, p = 0.005). Mothers who were not living in joint families had 1.9 (95% CI, 1.2, 2.9) higher odds of practicing EBF or predominant feeding for four months of age or beyond compared with those who were living in joint families (Table 5). Similarly, women who correctly answered when we asked how long only breast milk is enough for infants were practicing EBF or predominant feeding for longer periods of time compared with those who did not answer correctly. Infants who did not require hospitalization before four months of age had a 2.7 (95% CI, 0.96, 7.4) higher odds for practicing EBF or predominant breastfeeding compared with those who required hospitalization. However, mother's education status, occupation and perinatal conditions like types of delivery, birth weights were not significantly associated with EBF or predominant feeding for four months of age or beyond.

**Table 5:** Factors influencing exclusive or predominant breastfeeding in Bhaktapur , Nepal: logistic regression

Variable		Exclusive or predominant feeding for 4 months or beyond	Unadjusted	p value	**Adjusted**^1^	p value
	**N = 325**	**(n = 153)**	**Odds Ratio**		**Odds Ratio**	
			(95% CI)		(95% CI)	
		
**Knowledge about breastfeeding for 6 months**						
Yes	149	81(54%)	1			
No	176	72 (41%)	0.6 (0.4, 0.9)	0.01	0.6 (0.4, 0.9)	0.01
**Normal delivery**						
Yes	268	126 (47%)	1			
No	57	27 (47%)	1.0 (0.6, 1.8)	0.96	1.1 (0.6, 1.9)	0.89
**No work or only agricultural work**						
Yes	219	105 (48%)	1			
No	106	48 (45%)	0.9 (0.6, 1.3)	0.48	0.9 (0.5, 1.5)	0.48
**Mother with > grade 10 education**						
Yes	70	29 (41%)	1			
No	255	124 (49%)	1.3 (0.8, 2.3)	0.28	1.3 (0.7, 2.3)	0.36
**Children from joint families**						
Yes	205	85 (41%)	1			
No	120	68 (57%)	1.8 (1.2, 2.9)	0.008	1.9 (1.2, 2.9)	0.009
**Required hospitalization within first 4 months of life^2^**						
Yes	20	6 (30%)	1			
No	305	147 (48%)	2.2 (0.8, 5.8)	0.12	2.7 (1.0, 7.4)	0.05

^1 ^Odds ratio adjusted for the other listed variables

^2 ^Hospitalization for any illness (particularly pneumonia and diarrhea)

## Discussion

### Summary and key findings

Most of the infants in our study initiated breastfeeding within 24 hours and were fed with colostrum. However, it was very common to start herbal drops (*janamghuti*), water and semi-solid porridge as early as from the first months of life. The low prevalence of EBF at six months of age in our study (9%) was substantially lower than the 53% finding in the National Demographic Health Survey (NDHS) in 2006 [13], but in concordance with a recent study from India [19]. Although initiation of breastfeeding after birth, including the proportion who fed colostrum, were comparable to a study in Western Nepal, the prevalence of EBF in our study was lower [20].

The prevalence of EBF will also depend upon the methods of data collection and definitions used in the study. We asked in-depth questions on intake of other foods and drinks since birth [18]. The data on EBF in the NDHS survey was based on point estimation i.e. breastfeeding and complementary feeding status 24 hour prior to the interview in children under six months of age. Thus, children who were classified as EBF based on a 24 hour intake history could have taken foods at other times which were not recorded by the survey. This can substantially overestimate the prevalence of EBF [21]. Moreover, the breastfeeding pattern could be changed over time, if the interview was undertaken during early life. This was also discussed in a recent WHO consensus meeting on child feeding [17].

There are no traditional practices of prelacteal feeding in our study area. The reported prelacteal feedings were mostly given because of illnesses in the newborns or in the mother. The reported 42% who consumed nuts is surprising and is probably a peculiar practice in this community. Nuts, especially dates (*chokda*) and nutmegs (*jaiphal*) are mashed and given to infants. According to local belief it will sooth the baby and helps for normal sleep. Usually it is given in small amounts so it will not contribute with much energy.

Although breastfeeding is a social norm and universal practice in most communities, it is also a learned behavior and may be influenced by many factors including socio-economic, educational level and cultural [22]. "Insufficient breast milk" was the main reason for introducing other foods, especially semi-solid porridge (*lito*), before six months of age. This finding is concordant with another breastfeeding study conducted among employed women in peri-urban areas of Kathmandu [23], and a quantitative and qualitative study conducted among 750 young children residing in Far Western district of Baitadi, Nepal [24].

### Strength and limitations

Recruitment of infants was from a vaccine clinic and largely represented by infants from the local Newar ethnic community (78%). We believe that the infants in this study were representative of the local population, as our vaccine clinic is widely used in this community, and because we screened all infants during the study period [15]. Recall bias could be a source of error in this type of cross-sectional study, which can be overcome only by a prospective design. However, a prospective design will probably result in improvements in breastfeeding practices.

### Knowledge and breastfeeding practice

The mother's or caretaker's perception of "insufficient breast milk" is a well-known problem hindering optimal EBF practice in many communities [17,25]. It is interesting to note that some mothers could not mention any reason for early introduction of food, including semi-solid porridge, indicating that it is a common norm and spontaneous practice in this community. The findings of higher use of local herbal drops and early practice of partial feeding, leading to a significantly lower prevalence of EBF or predominant feeding among infants from joint families, indicates the importance of involvement of other family members during breastfeeding counseling, especially by mother-in-laws, who are directly involved in child care. In a typical joint family structure in Nepal, usually a mother does not have a decision-making role, so the feeding patterns of a child might be affected by the advice from other family members or relatives [20]. Our finding of higher rates of EBF or predominant feeding by mothers who are aware about appropriate age for EBF, indicates that dissemination of information on breastfeeding at the individual level is crucial [26]. There are abundant "state of the art" guidelines and materials from WHO and other organizations on how to promote EBF in the community. However, our findings indicate that these guidelines are not in use in our study area. One half of surveyed mothers could not tell how long a child should have only breast milk or mentioned that it would be sufficient for less than six months.

Breast milk has just the right amount of fat, sugar, water, and protein that is needed for a young baby's growth and development, and promotion of EBF practice has significant impact on child survival and mortality [6,27]. We found that not requiring hospitalization before four months of age was associated with longer EBF or predominant feeding with an OR of 2.7, however the 95% CI ranged from 0.96 to 7.1. Because of the nature of our study, we cannot draw any conclusions on causality based on the observed associations. For example, we cannot know whether hospitalization in early infancy caused lower prevalence of EBF, if it was the other way around, or if these two factors were caused by a third factor.

### Cultural beliefs

The cultural food taboos and beliefs which are deeply rooted in some communities are also found to be major factors influencing breastfeeding practices [10] in our setting. The readymade local herbal drops (*janamghuti*) were found to be a common tradition in our community, and infants usually started around one month of age. According to the local belief, these drops should be given to clean the stomach of infants and will remove unnecessary contents by inducing vomiting. It is also noteworthy to mention that in this local setting, rice is introduced at 5-6 months of age with a special ceremony called *Pasni*, or the rice feeding ceremony, which also seems to interfere with EBF for up to six months of age.

### Further work

The promotion of EBF could be done within the present healthcare framework during antenatal visits and/or vaccination clinics. All except seven women reported to have had at least one antenatal visit during their last pregnancy. Only one in four women reported that they received breastfeeding information during these antenatal visits. Unfortunately, we did not ask for more details on what type of information they had received. It is our impression that the antenatal visit in our setting focuses mainly on pregnancy, and there is no system or guidelines for breastfeeding education. The vaccination coverage in our study area is quite high (> 90% for all EPI vaccines) and comparable with the overall coverage at the district level [15], and represents an excellent opportunity for breastfeeding counseling, particularly during the first vaccine visit when the infant is around one month of age [19]. Future studies with prospective designs or focal group discussions should be carried out to clarify the extensive use of herbal drops as well as the early introduction of semi-solid food in this community.

## Conclusion

Despite the high proportion of women who initiated breastfeeding early after birth, the prevalence of EBF for up to six months of age was very low. Early introduction of other foods/drinks including semi-solid foods was common. Three quarters of the mothers did not receive any information on breastfeeding, indicating an urgent need for EBF promotion, which could be carried out within the existing healthcare system such as the antenatal and vaccination clinics.

## Competing interests

The authors declare that they have no competing interests.

## Authors' contributions

MU participated in the design, data collection and analysis, and wrote the first draft of the manuscript. RKC participated in design, analysis and manuscript preparation. PSS participated in the design of the study and manuscript preparation. LM participated in design, supervision and manuscript preparation. TAS participated in the design, statistical analysis and manuscript preparation. All authors read and approved the final manuscript.

## Abbreviations used

WHO: World Health Organization; EBF: Exclusive Breastfeeding; NDHS: National Demographic Health Survey.

## Supplementary Material

Additional file 1 **Questionnaire form used in the survey**. Click here for file
