# Supplementary material for: Infant feeding practices in Bhaktapur, Nepal: a cross-sectional, health facility based survey
Source: Int Breastfeed J. 2012 Jan 10;7:1. doi: 10.1186/1746-4358-7-1 (PMC3285083; doi:10.1186/1746-4358-7-1)
Supplement: Additional file 1 — Questionnaire form used in the survey. [file 1746-4358-7-1-S1.PDF]

Version 02.10.2007

BREAST FEEDING SURVEY FORM (Code 1=yes, 2=No, 9= Not applicable)

**BF_1**	Child Serial Number (बच्चाको क्रमसख्या)	BF_1
**BF_2**	Date (DMY, according to English calendar)<br>(अन्तर्वार्ता लिएको मिति अंग्रेजी क्यालेण्डर अनुसार)	BF_2
**BF_3**	FW code (फिल्ड वर्कर कोड)	BF_3
**BF_4**	Name of the child (बच्चाको नाम)	BF_4
**BF_5**	Age in months (महिनामा पुरा भएको उमेर)	BF_5
**BF_6**	Sex (1=Male, 2= Female) (१=पुरुष, २=महिला)	BF_6
**BF_7**	Birth order (जन्म क्रम)	BF_7
**BF_8**	Address (Tole, Ward and house number) ठेगाना (वडा र घर नम्बर)	BF_8
**BF_9**	Name of head of household (घरमुलिको नाम)	BF_9
**BF_10**	Age of mother in years (आमाका उमेर वर्षमा)	BF_10
**BF_11**	Did you get information on breastfeeding during ANC check up?<br>(गर्भवती जाँच गराउँदा स्तनपान बारे जानकारी पाउनुभयो?)	BF_11
**BF_12**	How long (months) do you think only breast milk will be enough for child ? (99 if do not know)<br>(तपाईको विचारमा कति महिनासम्म बच्चालाई आमाको दुधले मात्रै पुग्छ ?)	BF_12
**BF_13**	When was breastfeeding initiated after birth? (1=within 1 hour, 2=2-4 hours, 3= 5-24 hours, 4=after 24 hours)<br>बच्चा जन्मेपछि कहिले आमाको दुध ख्वाएको थियो ? १) एक घण्टाभित्र २) २-४ घण्टा, ३) ५ घण्टा - २४ घण्टा, ४) २४ घण्टापछि)	BF_13
**BF_14**	Which food was given first after birth (1=breast milk, 2= glucose water, 3=formula milk, 4=animal milk, 5=others, specify)<br>(बच्चालाई पहिलो खाना के दिएको थियो १= आमाको दुध, २=ग्लुकोज पानी ३=बोटल दुध ४=/गाई/भैसीको दुध, ५=अन्य)	BF_14
**BF_15**	Did you feed with colustrum ? (शुरुको पहेलो बाक्लो दुध दिनुभएको थियो ?)	BF_15
**BF_16**	How long child was feed on exclusively/predominately breastfeeding (months)?<br>(बच्चालाई आमाको दुध मात्रै कहिलेसम्म खुवाएको थियो ? (महिनामा लेख्नुहोस्)	BF_16
**BF_17**	If not breastfeeding, at what age breastfeeding stopped (months)?<br>(यदि आमाको दुध खुवाएको छैन भने, बच्चालाई कति महिनासम्म आमाको दुध खुवाएको थियो ?)	BF_17
**BF_18**	Reason for introducing other food before 6 months of age (1= crying/hungry, 2=mother illness, 3= no enough breast milk, 4=others)<br>(६ महिना अघि अरु खानेकुरा दिनाको कारण (१=भोकाएको/रोएको, २=आमाको रोग, ३= दुध नपुगेको, ४=अन्य)	BF_18
**BF_19**	Who gave advice for introduction of other food before 6 months of age (1=mother self, 2=maternal grand mother, 3 =paternal grand mother, 4=relatives/ neighbours,5=others.<br>(६ महिना अघि अरु खानकुरा दिन सल्लाह कसले दियो ?)१=आफै, २=बज्यै आमापटिको ३=बज्यै बुवापटिको, ४=छिमेकी ५=अन्य)	BF_19
**BF-20**	Was the child born at hospital/health facilities? (बच्चा अस्पताल/स्वास्थ्य संस्थामा जन्मेको हो?)	
**BF_21**	Type of delivery (1=normal, 2=cesarian section, operation, 3= vaccum, forcep)<br>(बच्चा जन्मेको किसिम) १=नरमल, २=अपरेशन, ३=भ्याकुम, फोरसेप)	
**BF_21.1**	Weight at birth (in kg) जन्मिन्दाको तौल (केजीमा)	.
**BF_22**	Weight (in kg) हालको तौल (केजीमा)	.
**BF_23**	Height / Length (in cm) लम्वाइ (सेन्टिमिटरमा)	.

When and how frequently introduced following foods? (1= regulary, 2= two/three times a week, 3= occassionaly)

	**Food items** (खानाको प्रकार)	Month of start	Month of stopped	Frequency of use
**BF_24**	Water (पानी)	24.1	24.2	24.3
**BF_25**	Janma Ghuti/ grape water (जन्मघुटी)	25.1	25.2	25.3
**BF_26**	Nuts/peanuts (काजु/बदाम)	26.1	26.2	26.3
**BF_27**	lito (लिटो) / Cerelac (सेरेलेक)	27.1	27.2	27.3
**BF_28**	Lactogen (ल्याक्टोजिन) / Tin milk (बट्टाको दुध)	28.1	28.2	28.3
**BF_29**	Animal milk (cow, buffalo, dairy) (गाई भैसीको दुध)	29.1	29.2	29.3
**BF_30**	Biscuits (बिस्कुट)	30.1	30.2	30.3
**BF_31**	Rice/Dal (भात/दाल)	31.1	31.2	31.3
**BF_32**	Vegetables, potato (सागसब्जी/आलु)	32.1	32.2	32.3
**BF_33**	Breads (रोटी/पाउरोटी)	33.1	33.2	33.3
**BF_34**	Fruits/fruit juice (फलफुल/फलफुलको रस)	34.1	34.1	34.3
**BF_35**	Noodles (चाउचाउ)	35.1	35.2	35.3
**BF_36**	Eggs (अण्डा)	36.1	36.2	36.3
**BF_37**	Meat (मासु)	37.1	37.2	37.3
**BF_38**	Beaten rice (चिउरा)	38.1	38.2	38.3
**BF_39**	Others, specify (अन्य भए उल्लेख गर्नुहोस)	39.1	39.2	39.3

	**Morbidity**	**Within 2 mo**	**3mon - 6mon**	**After 6 mon**
**BF_40**	Numbers of hopitalization (अस्पतालमा कति चोटी भर्ना भएको छ?)	39.1	39.2	39.3
**BF_40.1**	Specify, reason for hospitalization (अस्पतालमा भर्ना भएको कारण उल्लेख गर्नुहोस) ____________			
**BF_41**	Number of episodes of diarrhoea (कति पटक भाडापखाला लागेको छ?)	40.1	40.2	40.3
**BF_42**	Number of episodes of pneumonia (कति पटक न्यूमोनिया भएको छ?)	41.1	41.2	41.3
**BF_43**	Otitis media (कति पटक कान पाकेको छ?)	42.1	42.2	42.3
**BF_44**	Others, specify (अन्य भए उल्लेख गर्नुहोस)	44 text ____________		
**BF_45**	Child survey number (बच्चाको सर्भेक्षण नं.)	45		

**BF_46**	Living in rent (बहालमा बस्ने) (1=yes, 2==no)	BF_46
**BF_47**	Living in joint family (संयुक्त परिवार) (1=yes, 2==no)	BF_47
**BF_48**	Mother's occupation (1=no work, 2= agriculture, 3= Carpet worker, 4= daily wage earner, 5=self employs, 6=Services, 7=other) (आमाको पेशा) (१=काम नभएको, २=कृषि, ३=कार्पेट, ४=दैनिक ज्यालादारी, ५=निजी व्यवसाय, ६=कर्मचारी, ७=अन्य)	BF_48
**BF_49**	Father's occupation (पिताको पेशा)	BF_49
**BF_50**	Mother's education (1=illiterate, 2=primary, 3= up to class 10, 4= intermediate, 5= above) (आमाको शिक्षा कति छ ? १=अशिक्षित, २=साधारण, ३= कक्षा १०, ४=कलेज, ५=बढि)	BF_50
**BF_51**	Father's education (पिताको शिक्षा)	BF_51

Supervisor signature (सुपरभाइजरको सहि) ____________________
